# Identification of Robust Biomarkers for Early Predicting Efficacy of Subcutaneous Immunotherapy in Children With House Dust Mite-Induced Allergic Rhinitis by Multiple Cytokine Profiling

**DOI:** 10.3389/fimmu.2021.805404

**Published:** 2022-01-12

**Authors:** Shaobing Xie, Ruohao Fan, Qingping Tang, Xiao Cai, Hua Zhang, Fengjun Wang, Shumin Xie, Kelei Gao, Junyi Zhang, Zhihai Xie, Weihong Jiang

**Affiliations:** ^1^ Department of Otolaryngology Head and Neck Surgery, Xiangya Hospital of Central South University, Changsha, China; ^2^ Hunan Province Key Laboratory of Otolaryngology Critical Diseases, Xiangya Hospital of Central South University, Changsha, China; ^3^ National Clinical Research Center for Geriatric Disorders, Xiangya Hospital of Central South University, Changsha, China; ^4^ Department of Rehabilitation, Brain Hospital of Hunan Province, Hunan University of Chinese Medicine, Changsha, China

**Keywords:** allergic rhinitis, subcutaneous immunotherapy, biomarker, cytokine, children

## Abstract

**Background:**

Subcutaneous immunotherapy (SCIT) is an effective treatment for children with allergic rhinitis (AR), but its efficacy fluctuates among patients. There are no reliable candidate biomarkers for monitoring and predicting the response to SCIT. The present study aims to identify novel biomarkers for early predicting the efficacy of SCIT in pediatric AR patients based on multiple cytokine profiling.

**Methods:**

We prospectively recruited 72 children with house dust mite (HDM)-induced AR who were assigned to receive SCIT. The serum samples were collected and multiple cytokine profiling was conducted by Luminex assay at baseline. All patients were followed-up for 1 year and then categorized into effective and ineffective group based on their efficacy, and levels of 48 selected cytokines were tested and compared between the two groups. The potential cytokines were further validated by enzyme-linked immunosorbent assay (ELISA) in a cohort with 54 responders and 26 non-responders.

**Results:**

Sixty-nine of 72 children completed one-year follow-up schedule with 46 included in effective group and 23 in ineffective group. The results of multiple cytokine profiling showed that 15 cytokines (eotaxin, G-CSF, GM-CSF, IFN-γ, IL-12(p40), IL-13, IL-15, IL-16, IL-4, MIF, MIP-1α, RANTES, SCF, SDF-1α and VEGF) were dysregulated between effective and ineffective group (all P < 0.05). Unadjusted and adjusted multivariate analysis models highlighted that serum eotaxin, IFN-γ, IL-4 and MIF levels closely associated with the efficacy of SCIT in pediatric HDM-induced AR patients. In addition, receiver operating characteristic (ROC) curves revealed potential values of these four biomarkers in predicting the response to SCIT. Further ELISA validation results in the cohort of 80 pediatric patients demonstrated that serum eotaxin and IL-4 levels were elevated in responders while IFN-γ levels decreased in responders (all P < 0.05). ROC curves demonstrated that serum IL-4 exhibited more reliable accuracy in predicting SCIT efficacy than eotaxin and IFN-γ.

**Conclusion:**

Our discover–validation study suggested that cytokines including IL-4, eotaxin and IFN- γ may serve as robust biomarkers for early predicting response of SCIT in children with HDM-induced AR. These results strengthen the evidence that cytokines were associated with the response of SCIT and contributed to understand its underlying therapeutic mechanisms.

## Introduction

Allergic rhinitis (AR) is defined as a type I allergic disease caused by common aeroallergens such as pollen, dust mite and animal fur (1, 2). A recent epidemiological study based on 18 major cities of China reported that up to 17.6% respondents suffered from AR, and the prevalence increased gradually (3). Previous publications highlighted that house dust mite (HDM) was the most common allergen in perennial AR in southern regions of China (4, 5). As a common disease, AR is also prevalent in children and affects 10% to 30% of this population in the United States and other developed countries (6, 7). Accordingly, AR was closely associated with asthma and allergic conjunctivitis and, carried a heavy disease burden for children including attention and memory deficits, fatigue, sleep-deprived and even depression (8–10). Currently, available patient managements for AR include allergen avoidance, medications, and allergen-specific immunotherapy (AIT), and AIT is reported to be the only approach that can alter the natural course of this clinical disorder (11, 12). AIT can be performed by subcutaneous (SCIT) or sublingual (SLIT), and growing evidences proved that SCIT was better than SLIT in controlling allergic symptoms, improving compliance and reducing rescue medication consumption (13, 14). Although SCIT is a suitable treatment for pediatric AR, not all children respond to this therapy, and the efficacy always fluctuates across users (15–17). Therefore, to identify and validate potentially important laboratory predictors for SCIT response may play a pivotal role on SCIT treatment for pediatric AR patients.

Cytokines are small bioactive molecules secreted by a variety of cell types including immune cells and non-immune cells, and they are implicated in diverse pathophysiological processes such as tumor immunity, inflammation response hypoxia tolerance (18–20). Multiple cytokine profiling based on serum or urine specimen have been widely utilized in various clinical problems to explore biomarkers for early disease detection and prognosis prediction in malignant tumor, autoimmune diseases and inflammatory diseases (21–23). Previous studies have shown that peripheral cytokines were aberrantly expressed in AR patient and associated with its onset and/or disease severity (24, 25). In a recent study, Liu et al (26) evaluated the association between baseline laboratory parameters (including 10 cytokines) and clinical efficacy of SLIT in children with AR, and found that serum IL-10 and IL-35 levels linked to the response of SLIT. However, few is known on cytokine profiles in children with HDM-induced AR especially with regards to SCIT, and more efforts are urgently needed to explore predictors for SCIT response based on multiple cytokine profiling.

**Figure 1:**
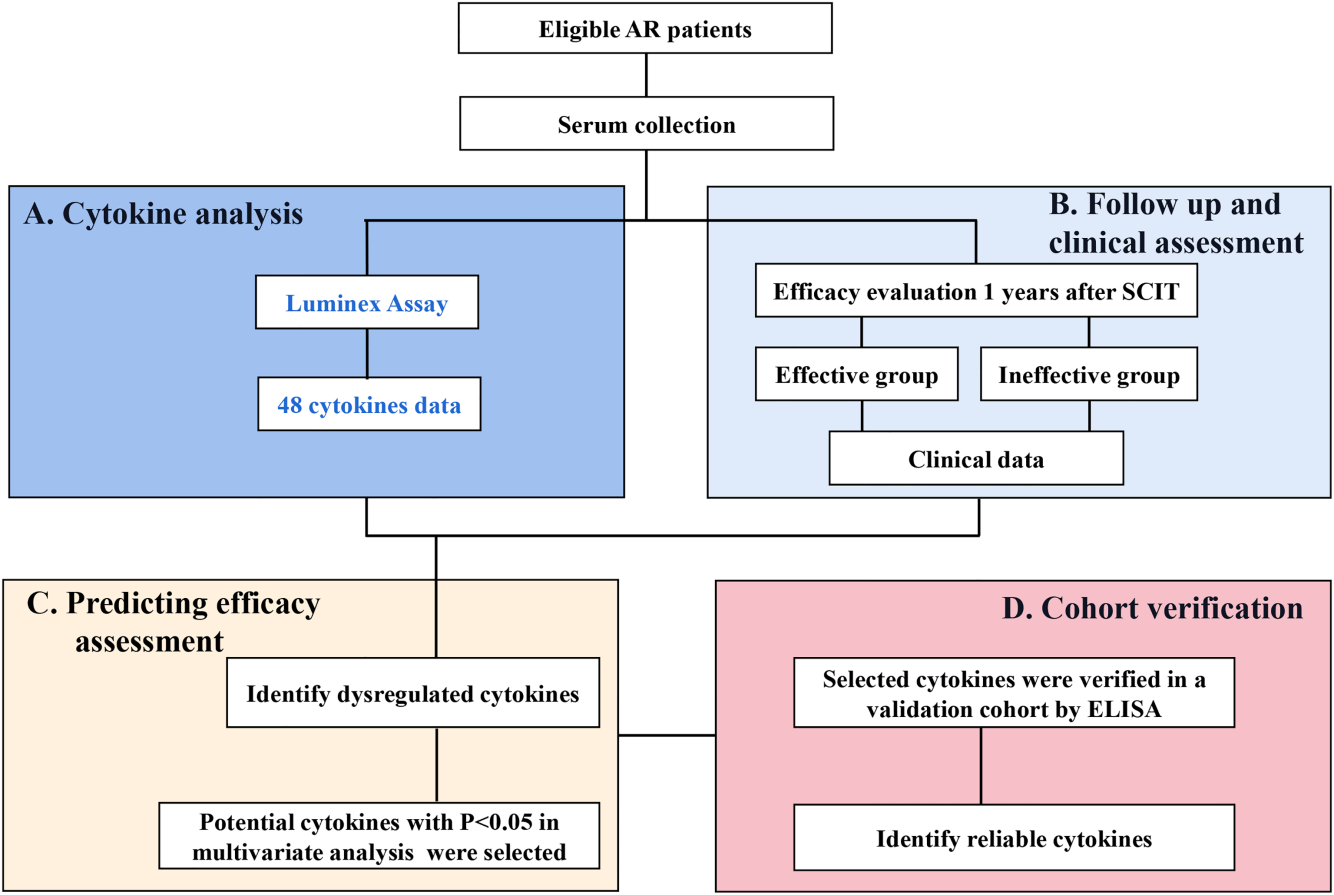
An overview of study profile for exploring serum predictive biomarkers for the efficacy of SCIT among pediatric HDM-induced AR patients. **(A)** multiple cytokines profiling analysis was conducted by Luminex assay; **(B)** follow-up and efficacy assessment; **(C)** cytokines levels were compared between effective group and ineffective group, and their predictive abilities were assessed. **(D)** potential cytokines were verified in a validation cohort by ELISA. HDM, house dust mite; AR, allergic rhinitis; SCIT, subcutaneous immunotherapy; ELISA, enzyme-linked immunosorbent assay.

Therefore, we hypothesized that some cytokines may serve as biomarkers predicting the SCIT response. To test this hypothesis, we asked two questions:1) what cytokines could be potentially important and 2) what cytokine are more reliable and accurate in predicting SCIT response in pediatric AR patients? To address these questions, we performed this discover and validation study and identify novel and reliable biomarkers for early predicting the efficacy of SCIT in pediatric AR patients based on multiple cytokine profiling. An overview of this study profile was showed in **Figure 1**.

## Materials And Methods

### Participants and Settings

We prospectively recruited 72 children with moderated to severe persistent HDM-induced AR who were assigned to receive SCIT between June 2019 and August 2019 at our medical center. All HDM-induced AR patients were diagnosed by allergy specialists referring to the allergic rhinitis and its impact on asthma (ARIA) guidelines (27). The criteria for patient selection included: 1) positive results of skin tests to Dermatophagoides farina (*Der f*) and/or Dermatophagoides pteronyssinus (*Der p*) (at least ++) and/or s-IgE level against *Der f* or *Der p* (>0.35 IU/mL); 2) age≥6 and ≤14 years old. The patients with acute exacerbation in asthma; with vasomotor rhinitis and other nasal or sinus diseases; a history of immunotherapy; with other inflammatory or septic diseases, or autoimmune diseases; consumption of anti-allergic drugs within 4 weeks before enrollment were excluded from thus study. Serum specimens were collected before onset of SCIT, and demographic and clinical data were recorded in **Table 1**. The Medical Ethics Committee of Xiangya Hospital of Central South University approved this study. All children guardians provided informed consent before the children were recruited.

**Table 1:** Demographics and clinical characteristics of patients between two groups.

Variables	Effective group (n=46)	Ineffective group (n=23)	P value
Sex			0.800
Male	26 (56.5%)	14 (60.9%)	
Female	20 (43.5%)	9 (39.1%)	
Age, years	9.6 ± 2.4	9.8 ± 2.4	0.749
BMI, kg/m^2^	17.9 ± 3.4	18.7 ± 3.5	0.385
Concomitant diseases			
Allergic asthma	11 (23.9%)	7 (30.4%)	0.573
Allergic conjunctivitis	6 (13.0%)	5 (21.7%)	0.487
Baseline VAS	5.7 ± 1.7	5.9 ± 1.6	0.616
Baseline TNSS	8.0 ± 1.8	8.2 ± 1.5	0.840

BMI, body mass index; TNSS, total nasal symptom score; VAS, visual analogue scale.

### Serum Samples Collection and Cytokine Measurements

The peripheral venous blood samples (5 mL from each patient) were obtained, and stored at room temperature for 1-2 hours, then the samples were centrifuged, and the supernatants were harvested and stored at -80°C for subsequent experiments. Serum multiple cytokine profiling was conducted by using multiplex assay kit (BioRad, CA, USA) according to the manufacturer’s instructions, and analyzed in a MAGPIX system (Luminex). The 48-Plex kit included the following cytokines: basic fibroblast growth factor (FGF), beta-nerve growth factor (β-NGF), cutaneous T cell attracting chemokine (CTACK), eotaxin, granulocyte colony stimulating factor (G-CSF), granulocyte-macrophage colony stimulating factor (GM-CSF), growth-regulated oncogene alpha (GRO-α), hepatocyte growth factor (HGF), interferon alpha-2 (IFN-α2), IFN-γ, interleukin (IL)-10, IL-12(p40), IL-12(p70), IL-13, IL-15, IL-16, IL-17, IL-18, IL-1α, IL-1β, IL-1ra, IL-2, IL-2R α, IL-3, IL-4, IL-5, IL-6, IL-7, IL-8, IL-9, interferon-inducible protein 10 (IP-10), leukemia inhibitory factor (LIF), monocyte chemotactic protein 1 (MCP-1), MCP-3, macrophage colony stimulating factor (M-CSF), macrophage migration inhibitory factor (MIF), monokine induced by interferon-gamma (MIG), macrophage inflammatory protein (MIP)-1α, MIP-1β, platelet-derived growth factor (PDGF)-BB, regulated on activation in normal T-cell expressed and secreted (RANTES), stem cell factor (SCF), stem cell growth factor- beta (SCGF-β), stromal cell-derived factor-1 alpha (SDF-1α), tumor necrosis factor- alpha (TNF-α), TNF-β, tumor necrosis factor related apoptosis inducing ligand (TRAIL) and vascular endothelial cell growth factor (VEGF). All cytokines and their detection limit were shown in **Table S1**. During data interpretation, cytokine values below the detection limit were imputed by utilizing robust regression on order statistics as previously described (28).

### Immunotherapy and Visit Schedule

All recruited patients were assigned to receive Novo-Helisen-Depot (NHD) allergen extracts (Allergopharma, Reinbek, Germany) which were derived from *Der f* and *Der p* at a 1:1 ratio. According to the manufacturer’s instructions, the conventional schedule of SCIT consists of build-up phase and maintenance phase. Build-up phase was initiated with the minimum dose with low concentration of NHD No.1 and was escalated to the maximum dose and high concentration NHD No.3, and the injection interval was generally between 7 and 14 days. For No.1 and No.2, the dose was increased to 0.1, 0.2, 0.4 and then 0.8 ml. For No.3, the dose was increased to 0.1, 0.2, 0.4, 0.6, 0.8 and then 1.0 ml. During the maintenance phase, patients were administered 1.0 ml of No. 3, and the injection interval was 4 to 6 weeks. SCIT was performed in the outpatient under the guide of allergy specialists, all children was observed for >30 minute before they leave the hospital for safety assessments. All adverse reactions were recorded during the whole visit schedule. The whole treatment course was recommended to last 3 to 5 years as previously described (29, 30).

### Follow-Up and Efficacy Evaluation

All participants were followed up for at least 1 year, and their symptom and drug consumption during the whole treatment were recorded. The clinical effectiveness of SCIT was judged according to the improvement of clinical symptoms and the reduction in the rescue drug consumption as our previous studies described. Briefly, symptom and medication score (SMS) was calculated as the sum of total nasal symptom score and final medication score in the previous week and recorded weekly. Patients who obtained more than 30% reduction of baseline SMS was regarded as effective, otherwise, SCIT was considered as ineffective (31, 32).

### Validation Cohort Recruitment and Cytokines Validation

For further confirmation of potential biomarkers in discovery cohort, another independent cohort consisting of 80 HDM-induced AR children who have been treated with SCIT for more than 1 year were enrolled. After early efficacy evaluation, 54 patients were regarded as effective and 26 patients were considered as ineffective after 1-year treatment and serum samples were harvested. The potential cytokines levels were detected by commercial enzyme-linked immunosorbent assay (ELISA) kits (Multisciences, Hangzhou, China) according to the manufacturer’s instructions.

### Statistical Methods

Numerical variable were described as the mean± standard deviation, Student’s t-test was applied when variables distributed normally, Mann-Whitney U test was performed when data distributed non-normally. Categorical data were expressed as frequencies and percentages, and the difference was compared by utilizing Chi-square test. Logistic regression analysis and receiver operating characteristic (ROC) curves were conducted to explore factors associated with the response of SCIT and evaluate the potential values in predicting the therapeutic efficacy. All statistical analyses were performed on SPSS statistics software version19.0 (IBM, Chicago, IL, USA). For all tests, P value below 0.05 for two-side was regarded as statistically significant.

## Results

### Baseline Data of All Subjects

In total, 69 patients finished 1 year of visit schedule and provided complete follow-up data, and 3 patients dropped out from this study. Among these patients, 46 of them were categorized into effective group, and 23 patients were included in ineffective group. Demographic and clinical data of all participants are listed in **Table 1**, and no statistic difference was observed in sex, age, body mass index (BMI), concomitant diseases rate, baseline visual analogue scale (VAS) and TNSS (all P > 0.05).

### Distinct Clustering of Cytokines Associated With SCIT Efficacy

**Figure 2:**
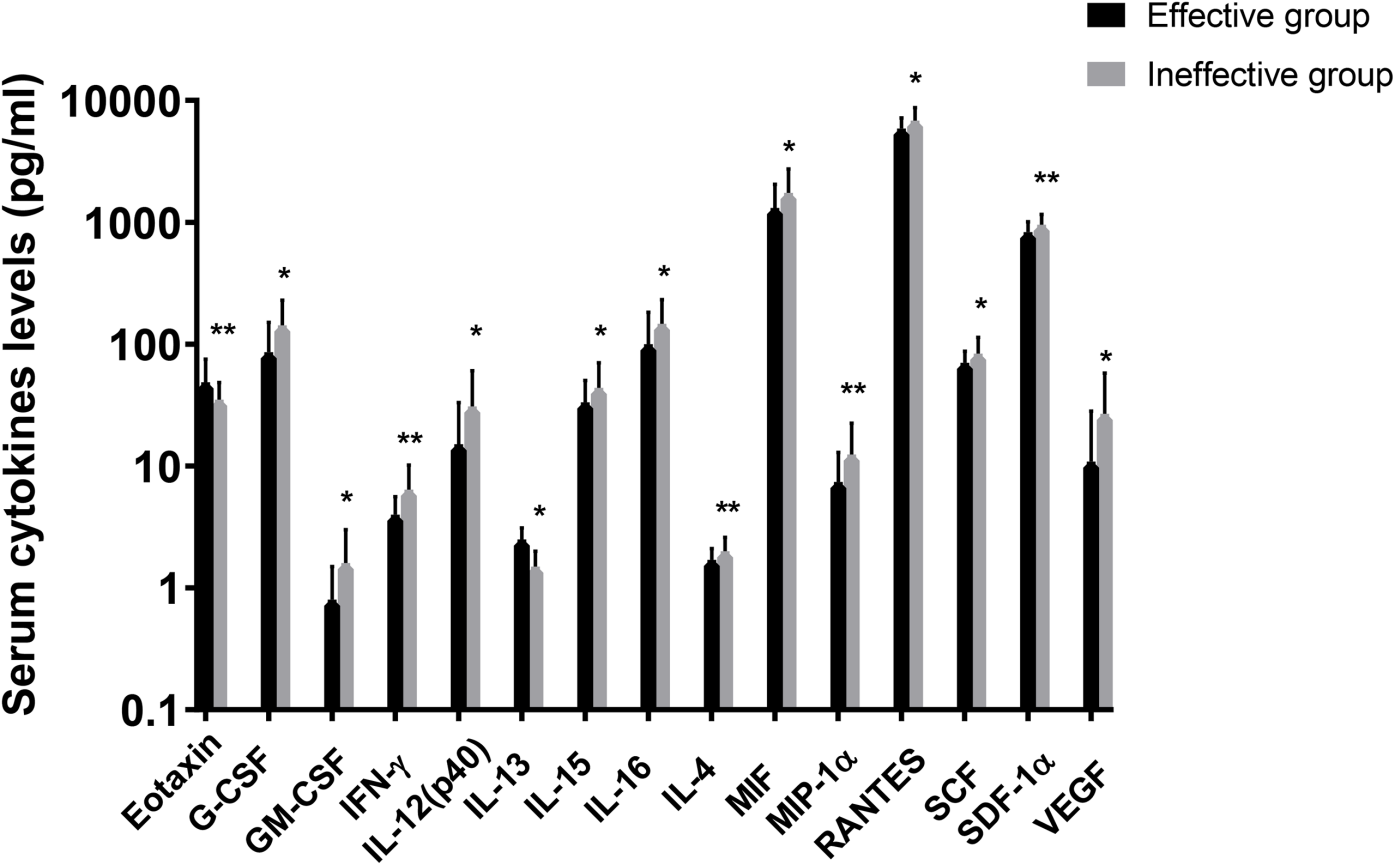
Logarithmic distribution of levels of 15 cytokines which were dysregulated between effective group and ineffective group. GM-CSF, granulocyte-macrophage colony stimulating factor; IFN, interferon; IL, interleukin; MIF, macrophage migration inhibitory factor; MIP, macrophage inflammatory protein; RANTES, regulated on activation in normal T-cell expressed and secreted; SCF, stem cell factor; SCGF, stem cell growth factor; SDF, stromal cell-derived factor; VEGF, vascular endothelial cell growth factor. *P < 0.05, **P < 0.01.

**Figure 3:**
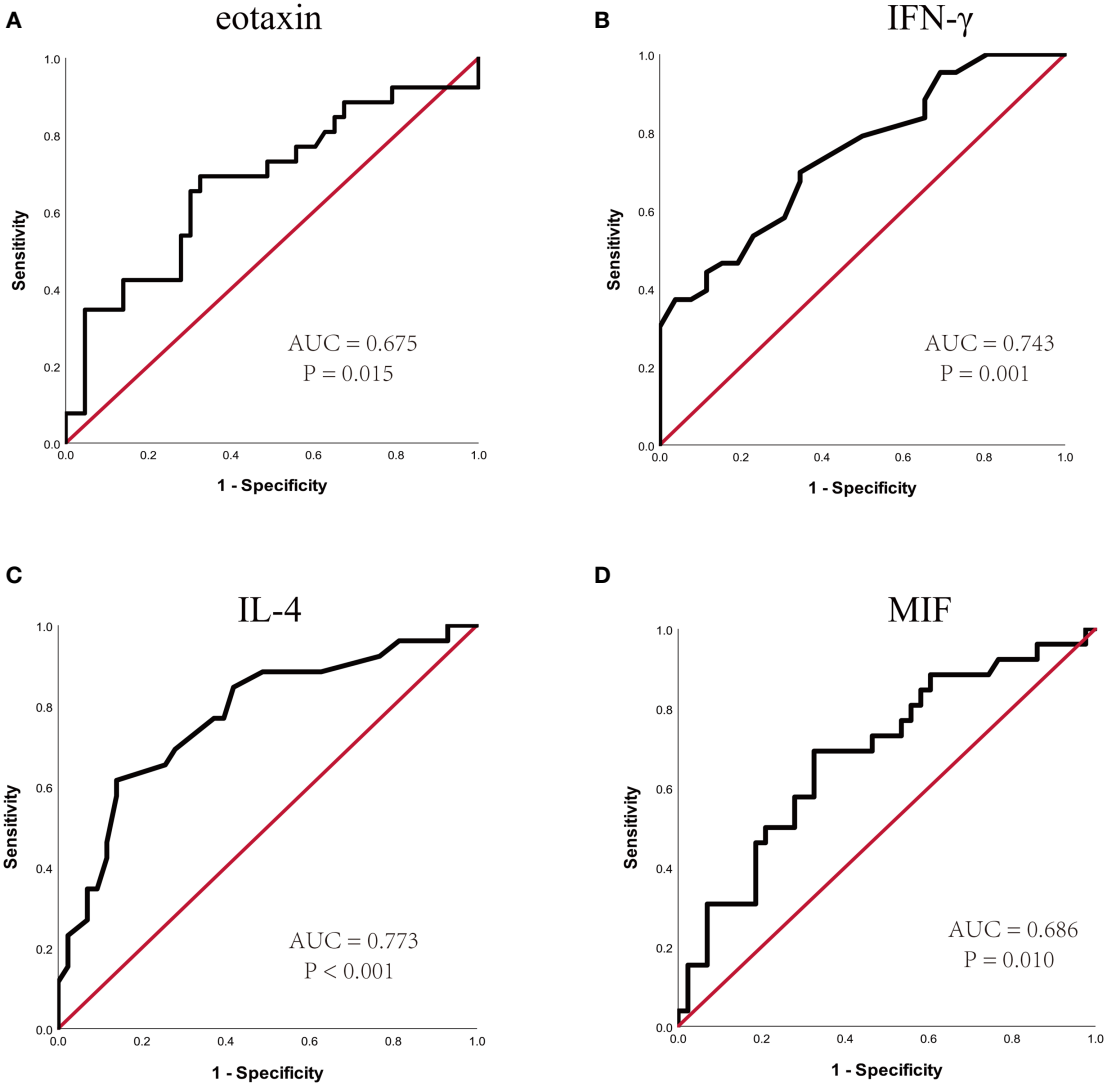
ROC curves of potential predictive biomarkers for the efficacy of SCIT among pediatric HDM-induced AR patients. **(A)** eotaxin; **(B)** IFN-γ; **(C)** IL-4; **(D)** MIF. HDM, house dust mite; AR, allergic rhinitis; SCIT, subcutaneous immunotherapy; ROC, receiver operating characteristics; AUC, area under the curve; IFN, interferon; IL, interleukin; MIF, macrophage migration inhibitory factor.

In the present study, 48 cytokines were detected in the serum samples, and their abbreviations and descriptive statistics were exhibited in **Table 2**. As illustrated in **Table 3** and **Figure 2**, multiple cytokines (15/48) (eotaxin, G-CSF, GM-CSF, IFN-γ, IL-12(p40), IL-13, IL-15, IL-16, IL-4, MIF, MIP-1α, RANTES, SCF, SDF-1α and VEGF) concentrations were aberrantly expressed between effective group and ineffective group (all P < 0.05). Unadjusted and adjusted multivariate analysis results highlighted that serum eotaxin, IFN-γ, IL-4 and MIF levels closely correlated with the efficacy of SCIT in pediatric AR (**Table 4**). ROC curves in **Figure 3** suggested that these four biomarkers exhibited potential values in predicting the response to SCIT, and detailed parameters were displayed in **Table 5**.

**Table 2:** Serum 48 cytokines, abbreviations, and their descriptive statistics in children with HDM-induced AR (pg/mL).

Cytokines	Abbreviation	Mean (± SD)	Range
Basic fibroblast growth factor	Basic FGF	27.1 ± 8.7	14.5-75.4
beta-Nerve growth factor	β-NGF	1.5 ± 1.8	0.2-6.8
Cutaneous T cell attracting chemokine	CTACK	987.5 ± 365.3	288.7-2147.0
Eotaxin	Eotaxin	43.6 ± 23.2	10.6-143.3
Granulocyte colony stimulating factor	G-CSF	107.6 ± 77.9	5.7-417.1
Granulocyte-macrophage colony stimulating factor	GM-CSF	1.1 ± 1.1	0.1- 6.4
Growth-regulated oncogene alpha	GRO-α	311.8 ± 113.5	13.7-486.9
Hepatocyte growth factor	HGF	376.5 ± 224.3	70.7-1387.0
Interferon alpha-2	IFN-α2	2.2 ± 2.1	0.0-7.2
Interferon gamma	IFN-γ	4.9 ± 2.9	1.7- 22.0
Interleukin-10	IL-10	2.2 ± 4.6	0.2-31.1
Interleukin-12(p40)	IL-12(p40)	30.9 ± 29.7	1.3-98.2
Interleukin-12(p70)	IL-12(p70)	1.5 ± 4.8	0.2-40.1
Interleukin-13	IL-13	1.4 ± 1.3	0.3-7.8
Interleukin-15	IL-15	37.2 ± 21.5	11.4-119.6
Interleukin-16	IL-16	117.5 ± 86.2	17.8-454.5
Interleukin-17	IL-17	6.7 ± 4.6	2.6-40.7
Interleukin-18	IL-18	48.8 ± 95.6	0.4-808.7
Interleukin-1 alpha	IL-1α	11.0 ± 11.8	1.3-94.3
Interleukin-1beta	IL-1β	2.2 ± 1.2	0.5-9.2
Interleukin 1 receptor antagonist	IL-1ra	539.2 ± 517.4	104.9-3358.0
Interleukin-2	IL-2	0.6 ± 0.6	0.1-4.3
Interleukin-2R alpha	IL-2R α	80.1 ± 62.6	9.8-479.4
Interleukin-3	IL-3	0.1 ± 0	0.0-0.2
Interleukin-4	IL-4	1.8 ± 0.5	0.9-3.4
Interleukin-5	IL-5	5.0 ± 16.9	0.4-102.5
Interleukin-6	IL-6	1.2 ± 3.4	0.1-20.0
Interleukin-7	IL-7	6.0 ± 5.6	0.8-26.6
Interleukin-8	IL-8	94.3 ± 96.1	2.2-489.7
Interleukin-9	IL-9	235.9± 23.2	172.5-286.1
Interferon-inducible protein 10	IP-10	432.7 ± 402.9	152.4-3215.0
Leukemia inhibitory factor	LIF	47.5 ± 16.0	20.5-111.5
Monocyte chemotactic protein 1	MCP-1	48.4 ± 40.7	4.1-230.9
Monocyte chemotactic protein 3	MCP-3	2.9 ± 6.7	0.1-40.6
Macrophage colony stimulating factor	M-CSF	25.9 ± 15.7	7.6-117.7
Macrophage migration inhibitory factor	MIF	1841.5 ± 993.5	213.7-4069.0
Monokine induced by interferon-gamma	MIG	271.1 ± 397.5	72.7-3253.0
Macrophage inflammatory protein-1 alpha	MIP-1α	9.3 ± 7.8	0.8-50.9
Macrophage inflammatory protein-1 beta	MIP-1β	179.1 ± 124.5	99.8-1128.0
Platelet-derived growth factor-BB	PDGF-BB	1968.2 ± 806.3	543.3-4893.0
Regulated on activation in normal T-cell expressed and secreted	RANTES	6234.3 ± 1533.2	3226.0-9925.0
Stem cell factor	SCF	75.3± 23.5	37.0-162.3
Stem cell growth factor- beta	SCGF-β	167253.5 ± 46810.5	40696.0-295946.0
Stromal cell-derived factor-1 alpha	SDF-1α	877.1 ± 197.3	526.4-1255.0
Tumor necrosis factor- alpha	TNF-α	19.4 ± 7.1	9.3-41.2
Tumor necrosis factor- beta	TNF-β	222.8 ± 22.7	156.4-263.1
Tumor necrosis factor related apoptosis inducing ligand	TRAIL	27.4 ± 7.8	4.9-60.9
Vascular endothelial cell growth factor	VEGF	16.8 ± 24.5	2.5-106.1

HDM, house dust mite; AR, allergic rhinitis; SD, standard deviation.

**Table 3:** Comparison of serum 48 cytokines levels between effective and ineffective group (pg/mL).

Cytokines	Effective group (n=46)	Ineffective group (n=23)	P value
Basic FGF	27.4 ± 10.0	26.5 ± 6.1	0.658
β-NGF	1.3 ± 1.7	1.9 ± 2.0	0.162
CTACK	926.9 ± 307.7	1087.7 ± 432.5	0.076
Eotaxin	48.8 ± 26.3	35.0 ± 13.4	**0.005**
G-CSF	86.1 ± 64.7	143.0 ± 85.9	**0.013**
GM-CSF	0.8 ± 0.7	1.6 ± 1.4	**0.018**
GRO-α	300.6 ± 104.3	330.3 ± 127.2	0.296
HGF	331.8 ± 150.2	450.4 ± 299.8	0.069
IFN-α2	0.9 ± 1.7	2.2 ± 2.1	0.070
IFN-γ	4.0 ± 1.6	6.4 ± 3.8	**0.004**
IL-10	2.2 ± 5.4	2.0 ± 3.0	0.821
IL-12(p40)	15.1 ± 18.1	30.9 ± 29.7	**0.019**
IL-12(p70)	0.7 ± 0.4	2.75 ± 7.7	0.203
IL-13	1.9 ± 1.0	1.4 ± 0.7	**0.029**
IL-15	33.3 ± 17.0	43.8 ± 26.5	**0.047**
IL-16	99.9 ± 82.6	146.6 ± 85.6	**0.028**
IL-17	6.7 ± 5.6	6.6 ± 2.2	0.942
IL-18	51.6 ± 120.3	44.2 ± 22.6	0.760
IL-1α	11.7 ± 14.5	9.8 ± 4.8	0.500
IL-1β	2.2 ± 1.4	2.3 ± 0.8	0.614
IL-1ra	474.3 ± 548.9	646.5 ± 450.4	0.182
IL-2	0.5 ± 0.3	0.8 ± 0.9	0.073
IL-2R α	84.9 ± 76.4	72.3 ± 27.3	0.424
IL-3	0.1 ± 0	0.1 ± 0	0.722
IL-4	2.0 ± 0.6	1.5 ± 0.5	**0.002**
IL-5	4.5 ± 15.2	5.8 ± 19.7	0.774
IL-6	0.9 ± 3.0	1.8 ± 4.0	0.263
IL-7	5.2 ± 5.1	7.3 ± 68.2	0.146
IL-8	82.1 ± 94.0	114.5 ± 97.9	0.177
IL-9	232.95 ± 22.0	240.7 ± 24.8	0.182
IP-10	422.2 ± 476.6	450.1± 243.9	0.783
LIF	46.4 ± 17.3	49.5 ± 13.9	0.441
MCP-1(MCAF)	42.2 ± 41.9	58.8 ± 37.0	0.100
MCP-3	2.2 ± 6.9	4.1 ± 6.2	0.257
M-CSF	25.8 ± 17.6	26.1 ± 12.1	0.950
MIF	1312.7 ± 737.8	1741.5 ± 993.5	**0.047**
MIG	308.2 ± 495.4	209.7 ± 105.9	0.322
MIP-1α	7.4 ± 5.5	12.4 ± 10.0	**0.008**
MIP-1β	157.0 ± 33.8	215.5 ± 195.0	0.141
PDGF-BB	1823.0 ± 704.6	2208.4 ± 915.4	0.054
RANTES	5868.5 ± 1326.3	6839.4 ± 1861.5	**0.010**
SCF	70.2 ± 17.2	83.8 ± 29.7	**0.041**
SCGF-β	168673.4 ± 40710.8	164905.2 ± 56272.1	0.749
SDF-1α	828.7 ± 181.0	957.2 ± 200.4	**0.008**
TNF-α	19.0 ± 7.0	20.0 ± 7.4	0.563
TNF-β	222.5 ± 22.5	223.3 ± 23.5	0.885
TRAIL	26.5 ± 8.0	28.7 ± 7.3	0.258
VEGF	10.9 ± 17.3	26.8 ± 31.1	**0.023**

FGF, fibroblast growth factor; NGF, nerve growth factor; CTACK, cutaneous T cell attracting chemokine; G-CSF, granulocyte colony stimulating factor; GM-CSF, granulocyte-macrophage colony stimulating factor; GRO, growth-regulated oncogene; HGF, hepatocyte growth factor; IFN, interferon; IL, interleukin; IP, interferon-inducible protein; LIF, leukemia inhibitory factor; MCP, monocyte chemotactic protein; M-CSF, macrophage colony stimulating factor; MIF, macrophage migration inhibitory factor; MIG, monokine induced by interferon-gamma; MIP, macrophage inflammatory protein; PDGF, platelet-derived growth factor; RANTES, regulated on activation in normal T-cell expressed and secreted; SCF, stem cell factor; SCGF, stem cell growth factor; SDF, stromal cell-derived factor; TNF, tumor necrosis factor; TRAIL, tumor necrosis factor related apoptosis inducing ligand; VEGF, vascular endothelial cell growth factor. Bold indicates statistical significance.

**Table 4:** Unadjusted and adjusted binary logistic regression exploring factors associated with SCIT efficacy.

Variables	Unadjusted		Adjusted	
	OR (95% CI)	P value	OR (95% CI)	P value
Eotaxin	2.154 (1.579-3.863)	**0.008**	1.313 (0.921-1.699)	**0.012**
G-CSF	1.028 (0.977-1.081)	0.288	1.033 (0.929-1.150)	0.547
GM-CSF	2.599 (1.375-5.627)	**0.024**	1.700 (0.095-6.279)	0.718
IFN-γ	0.411 (0.232-0.721)	**0.002**	1.845 (0.902-2.424)	**0.004**
IL-12(p40)	1.009 (0.967-1.053)	0.673	0.991 (0.942-1.042)	0.721
IL-13	2.398 (0.950-5.812)	0.086	4.837 (1.586-9.736)	**0.042**
IL-15	0.978 (0.927-1.032)	0.419	1.053 (0.961-1.155)	0.270
IL-16	0.994 (0.967-1.022)	0.694	0.999 (0.948-1.052)	0.957
IL-4	5.710 (1.892-32.867)	**0.041**	6.968 (1.922-42.934)	**0.028**
MIF	1.301 (1.007-1.922)	**0.018**	1.027 (1.005-1.625)	**0.022**
MIP-1α	0.846 (0.515-1.390)	0.509	0.700 (0.201-1.145)	0.575
RANTES	1.015 (0.968-1.061)	0.541	0.999 (0.978-1.021)	0.306
SCF	1.034 (0.984-1.086)	0.185	1.093(0.939-1.272)	0.253
SDF-1α	0.989 (0.978-1.001)	0.069	0.982 (0.982-1.007)	**0.034**
VEGF	0.992 (0.929-1.060)	0.821	0.097 (0.889-1.118)	0.958

SCIT, subcutaneous immunotherapy; OR, odds rate; CI, confidence interval; G-CSF, granulocyte colony stimulating factor; GM-CSF, granulocyte-macrophage colony stimulating factor; IFN, interferon; IL, interleukin; MIF, migration inhibitory factor; MIP, macrophage inflammatory protein; RANTES, regulated on activation in normal T-cell expressed and secreted; SCF, stem cell factor; SDF, stromal cell-derived factor; VEGF, vascular endothelial cell growth factor.

Adjusted for age, gender, BMI, baseline VAS and TNSS. Bold indicates statistical significance.

**Table 5:** ROC analysis results of different predictors for SCIT efficacy.

Variables	AUC (95% CI)	P value	cutoff value	sensitivity	specificity
Eotaxin (pg/mL)	0.675 (0.538-0.811)	**0.015**	39.5	0.692	0.674
IFN-γ (pg/mL)	0.743 (0.627-0.859)	**0.001**	4.3	0.698	0.654
IL-4 (pg/mL)	0.773 (0.656-0.890)	**<0.001**	1.9	0.615	0.860
MIF (pg/mL)	0.686 (0.555-0.816)	**0.010**	1317.5	0.692	0.674

ROC, receiver operating characteristics; SCIT, subcutaneous immunotherapy; AUC, area under the curve; CI, confidence interval; IFN, interferon; IL, interleukin; MIF, migration inhibitory factor. Bold indicates statistical significance.

### Validation of Cytokines by ELISA

**Figure 4:**
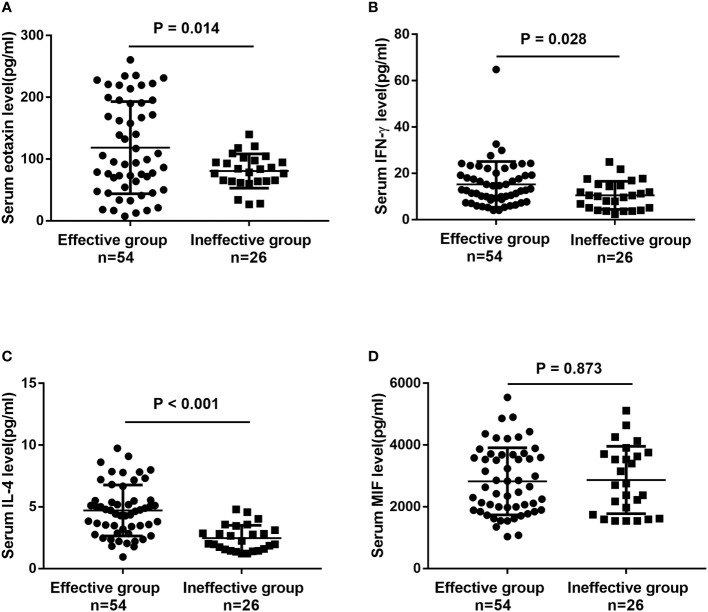
The serum levels of **(A)** eotaxin; **(B)** IFN-γ; **(C)** IL-4; **(D)** MIF between effective group and ineffective group in the validation cohort detected by ELISA. IFN, interferon; IL, interleukin; MIF, macrophage migration inhibitory factor; ELISA, enzyme-linked immunosorbent assay.

**Figure 5:**
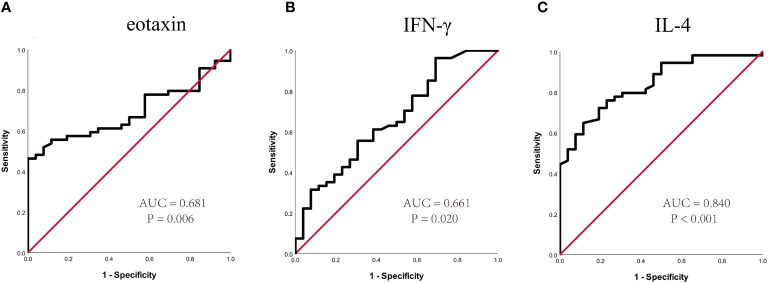
ROC curves of potential predictive biomarkers for the efficacy of SCIT in the validation cohort. **(A)** eotaxin; **(B)** IFN-γ; **(C)** IL-4. SCIT, subcutaneous immunotherapy; ROC, receiver operating characteristics; AUC, area under the curve; IFN, interferon; IL, interleukin.

To further confirm the above-mentioned results, serum levels of eotaxin, IFN-γ, IL-4 and MIF were measured in a validation cohort including 54 responders and 26 non-responders who have finished 1-year of SCIT. No statistic difference was found in demographic and clinical data between two groups (**Table S2**). The serum eotaxin and IL-4 levels were significantly enhanced in effective group than in effective group, while serum IFN-γ concentrations were lower in effective group (all P value < 0.05). No significant difference was observed in the serum MIF levels between two groups (**Figure 4**). Furthermore, ROC curves in **Figure 5** highlighted that serum IL-4 (AUC = 0.840, P < 0.001) exhibited stronger ability in predicting the efficacy of SCIT than serum IFN-γ (AUC = 0.661, P =0.020) and eotaxin (AUC = 0.681, P = 0.006), and the detailed parameters were shown in **Table S3**.

## Discussion

The efficacy of SCIT has been proved and widely administrated in clinical practice, but only a portion of patients benefit from this therapy (12, 14, 33, 34).An early and appropriate method to identify the patients who more likely respond to SCIT is urgently needed to conserve resources and achieve personalized treatment. Although previous studies found several biomarkers including serum periostin (35), specific IgE/total-IgE ratio (26) and (36), which might be associated with the efficacy of SCIT, no validated biomarkers predicting the SCIT response were available in clinical practice for patients with AR.

To address this issue, we conducted a discover–validation study with a combined application of multiplex assay and further ELISA to explore potential predictive biomarkers for the efficacy of SCIT in pediatric HDM-induced AR patients. Our principal findings include: 1) effective group of the 69 patients exhibited discriminative serum cytokines compared to ineffective group, and identified four potential predictors with high accuracy (eotaxin, IFN-γ, IL-4 and MIF); 2) furthermore, serum eotaxin, IFN-γ and IL-4 were validated to be predictive with reliable accuracy in a cohort of 80 patients. Taken together, these results indicated that serum eotaxin, IFN-γ and IL-4 were involved in the response of SCIT and its underlying therapeutic mechanisms in pediatric HDM-induced AR patients.

Eotaxin is a CC chemokine ligand (CCL) with diverse immunoreactivity, and composes of eotaxin-1 (CCL11), eotaxin-2 (CCL24), and eotaxin-3 (CCL26) (37–40). Previous studies reported that eotaxin was a pivotal mediator for promoting migration, activation and maturity of eosinophils, and high levels of eotaxin facilitated local and systemic eosinophils recruitment which were involved in the pathophysiology of various autoimmune and inflammatory diseases (41–43). A recent publication demonstrated that plasma eotaxin concentrations were elevated in chronic rhinosinusitis patients and eotaxin-3 levels positively associated with the degree of mucosal eosinophil infiltration, which suggesting eotaxin might be a major biomarker in upper airway inflammatory disease (44). Here, we firstly revealed that eotaxin levels were over-expressed in the serum of pediatric AR patients who responded to SCIT, which suggesting serum eotaxin might be concerned with the mechanisms of SCIT in AR. As is well known, type 2 inflammation and hyperactive eosinophilic response are predominated in the pathogenesis of AR and underlie the therapeutic mechanism of SCIT (14, 34, 45). Mechanistically, the immunotherapy has been demonstrated to regulate Th1/Th2 response balance and attenuate eosinophilic inflammation in AR, and up-regulation of Th1, regulatory B and T cells was considered prognostic (46, 47). Additionally, accumulating evidences indicated that a high baseline levels of Th2 immune and eosinophilic inflammation were beneficial in the process of allergen-specific immune tolerance (48–50). Therefore, we have reasons to believe that elevated serum eotaxin levels were conducive to development of immune tolerance during SCIT.

Another important finding was that pediatric HDM-induced AR patients in effective group presented higher baseline serum levels of IL-4 and lower IFN-γ than those in ineffective group, and serum IL-4 exhibited stronger ability in predicting the efficacy of SCIT than serum IFN-γ and eotaxin. IL-4 is a typical Th2 cytokine and IFN-γ is a representative Th1 cytokine, and they were widely accepted to be involved in the pathogenesis of AR (45, 51). It was established that type-2 cytokines such as IL-4, IL-5, or IL-13 were enhanced in AR and associated with allergic symptoms, and participated in the therapeutic mechanism of pharmacotherapy and AIT (52, 53). A recent study found that AIT could induce a decrease in CD4^+^ T cells and Th2 cytokine levels, and the change of cytokine levels closely related to the efficacy (54). Growing evidences showed that allergy tolerance induction characterized by the up-regulation of Treg and Breg cells were the important references of an effective AIT (55, 56). Regulatory cytokines secreted by Treg and Breg cells would dampen Th2 immune and eosinophilic inflammation and lead to a shift to Th1 cell response (54, 57). Therefore, we assumed that AIT only reduced Th2 cytokine levels to a limited extent when their baseline levels were low, and an excessive level of IFN-γ might contribute to a failure of Th1 blockade which suggesting a no response in AIT.

Some limitations should be addressed in our study. First, this study was limited to with a relatively small sample size in single medical center with the same ethnicity, which may raise the risk of selection bias and affect the generalization. Second, there is lack of internationally consensual criteria for evaluating efficacy of SCIT. Lastly, the period of follow-up was relatively short, which may weaken the conclusions. We will continue to track the all participant, and conduct further researches to confirm our conclusions in the future.

In conclusion, this is the first discover–validation study to apply multiple cytokine profiling to explore potential biomarkers for predicting the efficacy of SCIT in pediatric HDM-induced AR patients. We identified that serum eotaxin, IL-4 and IFN-γ might serve as robust biomarkers for early predicting response of SCIT in children with HDM-induced AR. These results strengthen the evidence that cytokines were associated with the response of SCIT and contributed to understand its underlying therapeutic mechanisms.

## Data Availability Statement

The raw data supporting the conclusions of this article will be made available by the authors, without undue reservation.

## Ethics Statement

The studies involving human participants were reviewed and approved by The Medical Ethics Committee of Xiangya Hospital of Central South University. Written informed consent to participate in this study was provided by the participants’ legal guardian/next of kin.

## Author Contributions

ShaX and RF wrote the manuscript. QT and XC performed data analysis. HZ, FW, and ZX collected sample. KG, ShuX and JZ provided statistical support. WJ designed the research study. All authors reviewed the manuscript and approved the final version.

## Funding

This work was supported by National Natural Science Foundation of China (No. 81770985, No. 81800917 and No. 81873695), Natural Science Foundation of Hunan Province (No.2020JJ4910).

## Conflict of Interest

The authors declare that the research was conducted in the absence of any commercial or financial relationships that could be construed as a potential conflict of interest.

## Publisher’s Note

All claims expressed in this article are solely those of the authors and do not necessarily represent those of their affiliated organizations, or those of the publisher, the editors and the reviewers. Any product that may be evaluated in this article, or claim that may be made by its manufacturer, is not guaranteed or endorsed by the publisher.
