# Supplementary material for: Identification of Robust Biomarkers for Early Predicting Efficacy of Subcutaneous Immunotherapy in Children With House Dust Mite-Induced Allergic Rhinitis by Multiple Cytokine Profiling
Source: Front Immunol. 2022 Jan 12;12:805404. doi: 10.3389/fimmu.2021.805404 (PMC8789884; doi:10.3389/fimmu.2021.805404)
Supplement: Supplementary file 1 [file Table_1.docx]

| Table S1 Serum 48 cytokines, abbreviations, and their detection limit (pg/mL) | | |
| --- | --- | --- |
| Cytokines | Abbreviation | Detection limit |
| Basic fibroblast growth factor | Basic FGF | 13.3-17330 |
| beta-Nerve growth factor | β-NGF | 0.62-3950 |
| Cutaneous T cell attracting chemokine | CTACK | 1.3-14902 |
| Eotaxin | Eotaxin | 0.06-3672 |
| Granulocyte colony stimulating factor | G-CSF | 3.08-234050 |
| Granulocyte-macrophage colony stimulating factor | GM-CSF | 0.34-16294 |
| Growth-regulated oncogene alpha | GRO-α | 27.06-88356 |
| Hepatocyte growth factor | HGF | 1.31-226440 |
| Interferon alpha-2 | IFN-α2 | 0.42-222378 |
| Interferon gamma | IFN-γ | 0.6-4632 |
| Interleukin-10 | IL-10 | 0.52-31536 |
| Interleukin-12(p40) | IL-12(p40) | 4.46-146618 |
| Interleukin-12(p70) | IL-12(p70) | 0.88-103398 |
| Interleukin-13 | IL-13 | 0.13-14748 |
| Interleukin-15 | IL-15 | 51.94-420160 |
| Interleukin-16 | IL-16 | 0.48-60976 |
| Interleukin-17 | IL-17 | 0.88-112216 |
| Interleukin-18 | IL-18 | 0.44-32684 |
| Interleukin-1 alpha | IL-1α | 1.58-51724 |
| Interleukin-1beta | IL-1β | 0.12-9340 |
| Interleukin 1 receptor antagonist | IL-1ra | 29.44-150288 |
| Interleukin-2 | IL-2 | 0.72-58186 |
| Interleukin-2R alpha | IL-2R α | 1.3-41144 |
| Interleukin-3 | IL-3 | 0.06-3492 |
| Interleukin-4 | IL-4 | 0.1-10404 |
| Interleukin-5 | IL-5 | 3.6-136870 |
| Interleukin-6 | IL-6 | 0.28-8160 |
| Interleukin-7 | IL-7 | 0.92-54966 |
| Interleukin-8 | IL-8 | 0.22-31100 |
| Interleukin-9 | IL-9 | 0.72-47820 |
| Interferon-inducible protein 10 | IP-10 | 0.52-31536 |
| Leukemia inhibitory factor | LIF | 2.38-141266 |
| Monocyte chemotactic protein 1 | MCP-1 | 0.56-16084 |
| Monocyte chemotactic protein 3 | MCP-3 | 0.46-2454 |
| Macrophage colony stimulating factor | M-CSF | 0.34-31854 |
| Macrophage migration inhibitory factor | MIF | 1.48-83270 |
| Monokine induced by Interferon-gamma | MIG | 0.68-59010 |
| Macrophage inflammatory protein-1 alpha | MIP-1α | 0.08-1826.77 |
| Macrophage inflammatory protein-1 beta | MIP-1β | 0.44-7678 |
| Platelet-derived growth factor-BB | PDGF-BB | 2.46-56248 |
| Regulated on activation in normal T-cell expressed and secreted | RANTES | 1.56-12432 |
| Stem cell factor | SCF | 0.64-68710 |
| Stem cell growth factor- beta | SCGF-β | 74.8-3477188 |
| Stromal cell-derived factor-1 alpha | SDF-1α | 1.4-58836 |
| Tumor necrosis factor- alpha | TNF-α | 1.88-159898 |
| Tumor necrosis factor- beta | TNF-β | 0.74-33522 |
| Tumor necrosis factor related apoptosis inducing ligand | TRAIL | 0.26-12682 |
| Vascular endothelial cell growth factor | VEGF | 10.32-72442 |
