# Supplementary material for: Identification of Robust Biomarkers for Early Predicting Efficacy of Subcutaneous Immunotherapy in Children With House Dust Mite-Induced Allergic Rhinitis by Multiple Cytokine Profiling
Source: Front Immunol. 2022 Jan 12;12:805404. doi: 10.3389/fimmu.2021.805404 (PMC8789884; doi:10.3389/fimmu.2021.805404)
Supplement: Supplementary file 2 [file Table_2.docx]

| Variables | Effective group  (n=54) | Ineffective group  (n=26) | P value |
| --- | --- | --- | --- |
| Sex |  |  | 0.810 |
| Male | 32 (59.3%) | 14 (53.8%) |  |
| Female | 22 (40.7%) | 12 (46.2%) |  |
| Age, years | 10.4 ± 2.5 | 10.3 ± 2.4 | 0.749 |
| BMI, kg/m^2^ | 18.1 ± 4.7 | 18.1 ± 2.9 | 0.958 |
| Concomitant diseases |  |  |  |
| Allergic asthma | 15 (27.8%) | 8 (30.8%) | 0.797 |
| Allergic conjunctivitis | 7 (13.0%) | 5 (19.2%) | 0.512 |
| Baseline VAS | 8.0 ± 1.8 | 7.8 ± 1.4 | 0.661 |
| Baseline TNSS | 5.7 ± 1.7 | 5.5 ± 1.5 | 0.749 |

Table S2 Demographics and clinical characteristics of patients in validation cohort

BMI, body mass index; TNSS, total nasal symptom score; VAS, visual analogue scale
