# Supplementary material for: Identification of Robust Biomarkers for Early Predicting Efficacy of Subcutaneous Immunotherapy in Children With House Dust Mite-Induced Allergic Rhinitis by Multiple Cytokine Profiling
Source: Front Immunol. 2022 Jan 12;12:805404. doi: 10.3389/fimmu.2021.805404 (PMC8789884; doi:10.3389/fimmu.2021.805404)
Supplement: Supplementary file 3 [file Table_3.docx]

| Variables | AUC (95% CI) | P value | cutoff value | sensitivity | specificity |
| --- | --- | --- | --- | --- | --- |
| Eotaxin (pg/mL) | 0.681 (0.5764-0.802) | 0.006 | 105.1 | 0.519 | 0.923 |
| IFN-γ (pg/mL) | 0.661 (0.494-0.734) | 0.020 | 12.1 | 0.556 | 0.692 |
| IL-4 (pg/mL) | 0.840 (0.755-0.926) | <0.001 | 5.2 | 0.963 | 0.308 |

Table S3 ROC analysis results of different predictors for SCIT efficacy in validation cohort

ROC, receiver operating characteristics; SCIT, subcutaneous immunotherapy; AUC, area under the curve; CI, confidence interval; IFN, interferon; IL, interleukin
